# Berberine inhibits NLRP3 Inflammasome pathway in human triple-negative breast cancer MDA-MB-231 cell

**DOI:** 10.1186/s12906-019-2615-4

**Published:** 2019-08-14

**Authors:** Mingjiang Yao, Xiaodi Fan, Bo Yuan, Norio Takagi, Sai Liu, Xiao Han, Junguo Ren, Jianxun Liu

**Affiliations:** 1 Xiyuan Hospital of China Academy of Chinese Medical Sciences, Institute of Basic Medical Sciences, No.1 Xiyuan Caochang, Haidian District, Beijing, 100091 China; 2 Key Laboratory of Pharmacology of Chinese Materia Medica, Beijing, 100091 China; 3 Laboratory of Pharmacology, School of Pharmacy, Faculty of Pharmacy and Pharmaceutical Sciences, Josai University, 1-1 Keyakidai, Sakado, Saitama, 350-0295 Japan; 4 Department of Applied Biochemistry, School of Pharmacy, Tokyo University of Pharmacy & Life Sciences, 1432-1 Horinouchi, Hachioji, Tokyo, 192-0392 Japan

**Keywords:** Berberine, Triple-negative breast cancer, NLRP3 inflammasome, Anti-inflammation

## Abstract

**Background:**

Breast cancer is still the most common malignant tumor that threatens the female’s life in the world, especially triple-negative breast cancer (TNBC), one of the most difficult subtypes. Lack of targeted therapies brings about urgent demand for novel treatments. In this study we aim to investigate the anti-tumor activity of Berberine (BBR), a Chinese plant-derived alkaloid, against the TNBC cell line MDA-MB-231 and elucidate its mechanism referring to anti-inflammation.

**Methods:**

Cell inhibition rate was measured by Cell Proliferation Assay, the cytotoxic effects was detected by Lactate dehydrogenase (LDH) leakage assay, the colony formation and migration potential were evaluated by colony formation assay and wound healing assay, the release of inflammatory cytokines was detected by EMD multifactor detection, and alterations of proteins and genes related to the NLR family pyrin domain containing 3 (NLRP3) inflammasome pathway were analyzed using western blotting and real-time Polymerase Chain Reaction (PCR).

**Results:**

BBR reduce the viability of MDA-MB-231 cells and increased the release of LDH from the cells in a dose-dependent manner, with and inhibition of colony formation potential and migration of the cells. BBR also caused a marked reduction in the secretion of proinflammatory cytokines, Interleukin-1α (IL-1α), Interleukin-1β (IL-1β), Interleukin-6 (IL-6), and tumor necrosis factor-α (TNF-α). Besides, a down-regulated behavior was observed with the expression of P2X purinoceptor 7 (P2X7), NLRP3, pro-caspase-1, apoptosis-associated speck-like protein containing a caspase-activation and recruitment domain (ASC), caspase-1 p20, Interleukin-18 (IL-18), IL-1β proteins and NLRP3, Caspase-1 and ASC mRNAs in the NLRP3 inflammasome cascade.

**Conclusions:**

Our results confirmed that BBR can effectively affect both tumor outgrowth and spontaneous metastasis in TNBC, and that we identified a new mechanism associated with inhibition the NLRP3 inflammasome pathway, suggesting its potential therapeutic relevance in clinical use.

## Background

Breast cancer is reported to be one of the most prevalent malignancies among women throughout the world, with a youth oriented tendency and continuously increasing mortality rate every year [1–3]. Despite considerable developments in anti-cancer therapies such as hormone therapy, chemotherapy, and targeted therapy that have improved the outcomes of breast cancer patients distinctly [4], there are still hurdles and challenges we should face in order to promote therapeutic efficacy, overcome side-effects and drug resistance, especially in triple-negative breast cancer (TNBC), a very aggressive subtype of breast cancer, possesses less than 30% of five-year survival rate in cases with metastasis [5, 6]. Herein, looking for efficient and safer therapeutic agents and thus investigate the pharmacological mechanism is critically needed for the treatment of TNBC.

Accumulating data indicate that inflammation is closely related with tumor initiation, progression and metastasis, and to a great extent due to its participating in the interaction between malignant cells and their microenvironment [7, 8]. On the one hand, there would be a series of pro-inflammatory mediators, for instance, the cytokines, chemokines and transcriptional factors secreted from the cancerous cells, on the other hand, these critical inflammation-related components, in turn, were proved to be the core molecular players in regulating signaling pathways and processes involved in oncogenesis [9]. Thus, regulation of inflammatory reaction is a promising anti-tumor strategy that should be paid more attention to.

The NLR family pyrin domain containing 3 (NLRP3) inflammasome, composed by NLRP3 oligomers and apoptosis-associated speck-like (ASC) adapter protein, is a key innate immune pathway through triggering the activation of caspase-1, leading to the processing of interleukin-1 beta (IL-1β) and IL-18, as well as inducing of pyroptosis and malignant transformation [10, 11], and have been proved to take part in the genesis and development of several inflammatory disorders, including inflammatory bowel disease (IBD) [12], pancreatitis [13], and may also increase the risk of cancer [14, 15]. Therefore, targeting the NLRP3 inflammasome pathway has opened the door for novel strategy in cancer prevention and treatment.

Berberine (BBR) is a kind of organic heteropentacyclic compound and natural isoquinoline alkaloid isolated from several traditional Chinese herbal plants as *Coptis chinensis (Huanglian)* and *Cortex Phellodendri Chinensis (Huangbai)*, which have been used to treat gastroenteritis caused diarrhea for more than one thousand years. BBR is an odorless yellow crystalline powder with characteristic alkaloid bitterness. Because of its poor solubility in water, the chloride or sulfate of BBR are more commonly used in practice due to the relatively water-soluble of the salt form [16]. A substantial body of evidence supports the multiple novel biological roles of BBR in anti-pathogenic microorganism [17], anti-diabetes [18], cerebrovascular protection [19, 20], and blood lipid regulation [21], among which the anti-inflammation effect is considered to be the most representative one and its molecule mechanisms have been revealed referring to certain signaling pathways and inflammatory factors [22, 23]. In our previous study [23], BBR has been found to inhibit oxidized low-density lipoprotein (ox-LDL) induced inflammation in J774A.1 cells by activating autophagy via the 5′ AMP-activated protein kinase (AMPK) / mechanistic Target of Rapamycin (mTOR) signaling pathway, also further proved the anti-inflammation effect of BBR. Recently, the anti-tumor activities of BBR have been revealed in various human cancer cells [24–26], and a broad range of mechanism are involved as inducing autophagy, apoptosis [27, 28], generation of reactive oxygen species [29] and cell cycle arrest [28]. Moreover, several studies have revealed that BBR could regulate NLRP3 inflammasome pathway in different types of macrophages, such as THP-1, J774A.1 and RAW264.7 [30–32]. However, whether BBR can affect the human breast cancer cell through inflammation related pathways has rarely been investigated before.

In this study, we identified a new potential mechanism by which BBR inhibits the growth of human breast cell line MDA-MB-231 associated with inhibition of the NLRP3 inflammasome pathway. This suggests that BBR may regulate inflammation in breast cancer, and could therefore have potential therapeutic relevance.

## Materials and methods

### Materials

Berberine Hydrochloride was purchased from National Institute for Food and Drug Control (China, Lot: 110713–201,212), dissolved in distilled water and filtered by a 0.22 μm filter. DMEM medium and Fetal Bovine Serum (FBS) were purchased from GIBCO® by Life Technologies (Carlsbad, CA, USA). The CellTiter 96® AQueous One Solution Cell Proliferation Assay (MTS) (Lot: 0000185040) was purchased from Promega Corp. (Madison, WI, USA). The LDH Cytotoxicity Assay Kit (Lot: 072418181030) was acquired from Beyotime Biotechnology Co., Ltd. (Shanghai, China). The Giemsa stain solution was provided by Solarbio Life Sciences (Beijing, China). The Cytokine Magnetic Bead assay kit (#RECYTMAG-65 K) was purchased from Millipore Corp. (Billerica, MA, USA). Antibodies used in this study were P2X7 from Bioss (Beijing, China); pro-Caspase-1, ASC, NLRP3, Caspase-1 p20 and IL-18 from Wanleibio (Shenyang, Liaoning, China); IL-1β from Proteintech (Wuhan, Hubei, China); β-actin from Cell Signaling Technology (Boston, MA, USA). ReverTra Ace qPCR RT Master Mix were purchased from Toyobo CO., LTD. (Osaka, Japan). TRIzol Reagent and ABI Power SYBR Green PCR Master Mix were purchased from Thermo Fisher Scientific, China (Shanghai, China). Primers used in this study were NLRP3, Caspase-1, and IL-1β, GAPDH from iGenebio CO., LTD. (Guangzhou, Guangdong, China).

### Cell culture and BBR treatment

MDA-MB-231 cells were obtained from Procell Life Science & Technology Co., Ltd. (Wuhan, Hubei, China) (CL-0150). Cells were cultured at a density of 1.0 × 10^5^ cells/ml in DMEM medium supplemented with 10% FBS and 100 U/ml of penicillin and 100 μg/ml of streptomycin at 37 °C in a humidified atmosphere (5% CO_2_ in air) and allowed to attach for 24 h. Then the cells were treated with indicated concentrations of BBR for another 48 h.

### Cell viability assay

After treatment with various concentrations of BBR (2.5, 5, 10, 20, 40, 60, 80, 100 μg/ml) for 48 h, the cell viability was measured by the CellTiter 96® AQueous One Solution Cell Proliferation Assay (MTS) according to the method previously described with mild modification [33]. Briefly, the MTS solution was added into 5 times volume of serum-free medium to get reaction solution, the supernatants in each well was discarded and the cells were gently rinsed once by phosphate-buffered saline (PBS), and then 120 μl of reaction solution was added into each well, after incubation at 37 °C for 2 h, the absorbance at 490 nm was measured with a microplate reader (Synergy™ 4, BioTek). The relative cell viability was expressed as the ratio of the absorbance of each treatment group against those of the corresponding untreated control group. The results were representative of three independent experiments and the IC_50_ values of BBR were calculated by GraphPad Prism® 6 software.

### Lactate dehydrogenase (LDH) cytotoxicity assay

MDA-MB-231 cells were cultured in 96-well plates, and the culture medium containing various concentration of BBR (10, 20, 40 μg/ml) were added to each well and incubated for 48 h. Then LDH leakage was measured using Cytotoxicity Detection Kit according to the manufacturer’s procedure. Briefly, 120 μl culture supernatants were collected from each well and then 60 μl freshly prepared reaction buffer supplied in the kit was added. 30 min after gentle mixing at room temperature, the absorbance at 490 nm for each sample was measured using a microplate reader (Synergy™ 4, BioTek). The release of LDH was represented as the folds of control group. The results were from three independent experiments.

### Colony formation assay

The reproductive viability of MDA-MB-231 cells was measured by colony formation assay as described previously [34, 35]. Briefly, cells were seeded into 6-well plates with the density of 1000cells/well, 24 h after cells had attached, the medium was changed by different concentrations of BBR contained medium and treated for another 48 h, then the medium was again replaced by fresh drug free medium and followed by another 12 days at 37 °C in a humidified atmosphere (5% CO_2_ in air). After that, the colonies were fixed with 100% methanol for 2 min and stained with Giemsa stain solution for 20 min in room temperature. Stained cell colonies in each well were flat scanned and analyzed, and the results were representative of three independent experiments.

### Wound healing assay

An in vitro wound healing assay was performed to evaluate the effect of BBR on cell migration followed by the methods previously described [36]. Briefly, MDA-MB-231 cells were seeded at a density of 1 × 10^5^ cells/ml in 24-well plates and allowed to form a confluent monolayer after 24 h. The layer of cells was then scraped with a 20-200 μL micropipette tip to create a wound of about 500 μm width, and then the cells were gently rinsed twice by PBS, followed by the treatment with indicated concentrations of BBR for 48 h. The cells were photographed at 0 h, 24 h and 48 h using an inverted microscope (IX51, Olympus, Japan) mounted with a digital camera (E330, Olympus, Japan). The distance between the edges of the cell-free areas was measured and the cell migration was calculated using the following equation: %R = [1-(wound length at T_24h (or 48h)_/wound length at T_0h_)] × 100% where %R is the recovery percentage, T_0h_ is the wound length at 0 h, T_24h_ and T_48h_ is the wound length at 24 h and 48 h after injury, respectively. The results were representative of three independent experiments.

### Inflammatory cytokines detection

The concentration of cytokines, TNF-α, IL-1α, IL-1β, and IL-6, in the supernatant of MDA-MB-231 cells after 48 h of BBR treatment was quantified by the EMD Millipore’s MILLIPLEX MAP Human Cytokine/Chemokine Magnetic Bead assay according to the manufacturer’s instructions. The median fluorescence intensity was assayed on a FLEXMAP 3D™ system, and a five-parameter logistic method was performed to obtain the fitting curve and thus calculate the concentration of cytokines in the supernatant samples.

### Quantitative real-time PCR

Total mRNA extraction and reverse transcription were prepared according to the method reported previously with slight modification [35, 37]. Briefly, total mRNA was extracted from cells by TRIzol Reagent and then quantified by SMA-1000 Micro-volume Spectrophotometer (Merinton Instrument Inc., Beijing, China). Complementary DNA was reverse-transcriptional synthesized from 1 μg of mRNA using ReverTra Ace® qPCR RT Master Mix according to the manufacture’s protocol by T-GRADIENT PCR Instrument (Biometra, Überlingen, Germany). Quantitative real-time PCR assay was performed using Applied Biosystems StepOnePlus™ Real-Time PCR System (Thermo Fisher Scientific, CA, US). Primers used in the study were: GAPDH (forward 5′-GATCATCAGCAATGCCTCCT-3′ and reverse 5′-TGTGGTCATGAGTCCTTCCA-3′), NLRP3 (forward 5′-AGGAAAAGGAAGGCCGACAC-3′ and reverse 5′-TGGAAGTGAGGTGGCTGTTC-3′), Caspase-1 (forward 5′-GAACTGCCCAAGTTTGAAGG-3′ and reverse 5′- AGCATCATCCTCAAACTCTTCTG-3′), and IL-1β (forward 5′-TCTGTACCTGTCCTGCGTGT-3′ and reverse 5′-ACTGGGCAGACTCAAATTCC-3′). The amplification conditions were: 10 min at 95 °C for initial denaturation; 40 reaction cycles including a denaturation step at 95 °C for 15 s with an annealing/extension step at 60 °C for 1 min; followed with an instrument preset melting curve analysis process. A fold change in relative expression of target gene was calculated using the comparative Ct (2^-ΔΔCt^) method.

### Western blot analysis

Total protein extraction and western blot analysis meet the previously described methods [35]. The collected cell pellets of each treated group were lysed in Leammli buffer with protease inhibitor, and then were centrifuged at 12000 rpm for 12 min at 4 °C. The supernatant was then extracted, of which the concentration was determined by Bradford (BIO-RAD, USA). Whole cell lysate were electrophoresed on 10% or 12% SDS-PAGE, and then the proteins were transferred to a polyvinylidene difluoride (PVDF) membrane (Millipore Corp, MA, USA), the levels of targeted proteins were detected using the following primary antibodies and dilution ratios: P2X7 (1:1000), NLRP3(1:500), pro-caspase-1(1:500), ASC (1:500), caspase-1 p20(1:500), IL-18(1:500), IL-1β(1:1000), and β-actin (1:2000). Blotted protein bands were detected with respective horseradish peroxidase-conjugated secondary antibody (1:20000) and an enhanced chemiluminescence (ECL) reagent (Thermo Fisher Scientific Inc., USA). Images were generated using ChemiDoc XRS+ System (BIO-RAD, USA). The visualized protein bands were quantified with Gel-Pro 4.0 software.

### Statistical analysis

All experiments were performed at least three times, the data were represented as means ± SD, and the statistical significance was analyzed by SPSS 19.0 using one-way ANOVA followed with Tukey’s Post Hoc analysis, the level of *p* < 0.05 was considered significant.

## Results

### Antiproliferative and cytotoxic effects of BBR

**Fig. 1:**
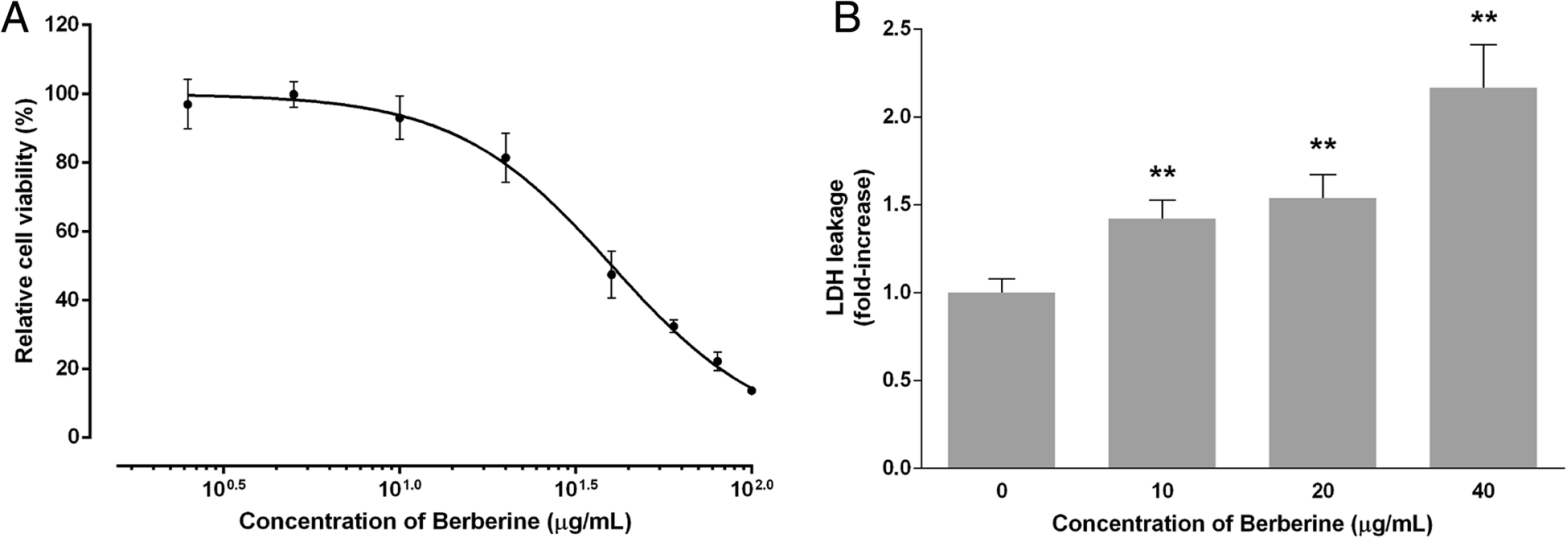
Effect of BBR on MDA-MB-231 viability and LDH release. Cells were seeded in 96-well plates at 5000 cells per well and treated with various concentrations of BBR for 48 h, the control wells were treated with equivalent amount of medium alone. The effect of BBR on MDA-MB-231 (**a**) viability and (**b**) LDH release was determined by the assays described in the Methods section. The relative cell viability and LDH leakage was calculated as the ratio of the absorbance at 490 nm of each treatment group against those of the corresponding untreated control group. Each value represents the mean ± S.D. (*n* = 6) of three independent experiments. * *p* < 0.05, ** *p* < 0.01, compared to the control

First, we examined the growth inhibitory effects of BBR using the MTS cell proliferation assay in human breast cancer cell line MDA-MB-231. A significant decrease in cell viability was observed after treatment with various concentrations of BBR (2.5, 5, 10, 20, 40, 60, 80, 100 μg/ml) for 48 h in a dose-dependent manner (96.98 ± 6.98%, 99.80 ± 3.69% 93.05 ± 6.38%, 81.43 ± 7.09%, 47.47 ± 6.79%, 32.38 ± 1.84%, 22.20 ± 2.75% and 13.67 ± 1.07%, respectively) with an IC_50_ of 40.06 μg/ml (Fig. 1a). We further evaluated the cytotoxic activity of BBR using LDH measurement assay. An increased LDH leakage in cell culture medium with increasing concentration of BBR was observed (1.00 ± 0.08 VS 1.42 ± 0.11, 1.54 ± 0.14, and 2.16 ± 0.24, respectively) (Fig. 1b), indicating BBR damaged the integrity of cell plasma membrane.

### BBR decreases colony formation potentials of MDA-MB-231 cells

**Fig. 2:**
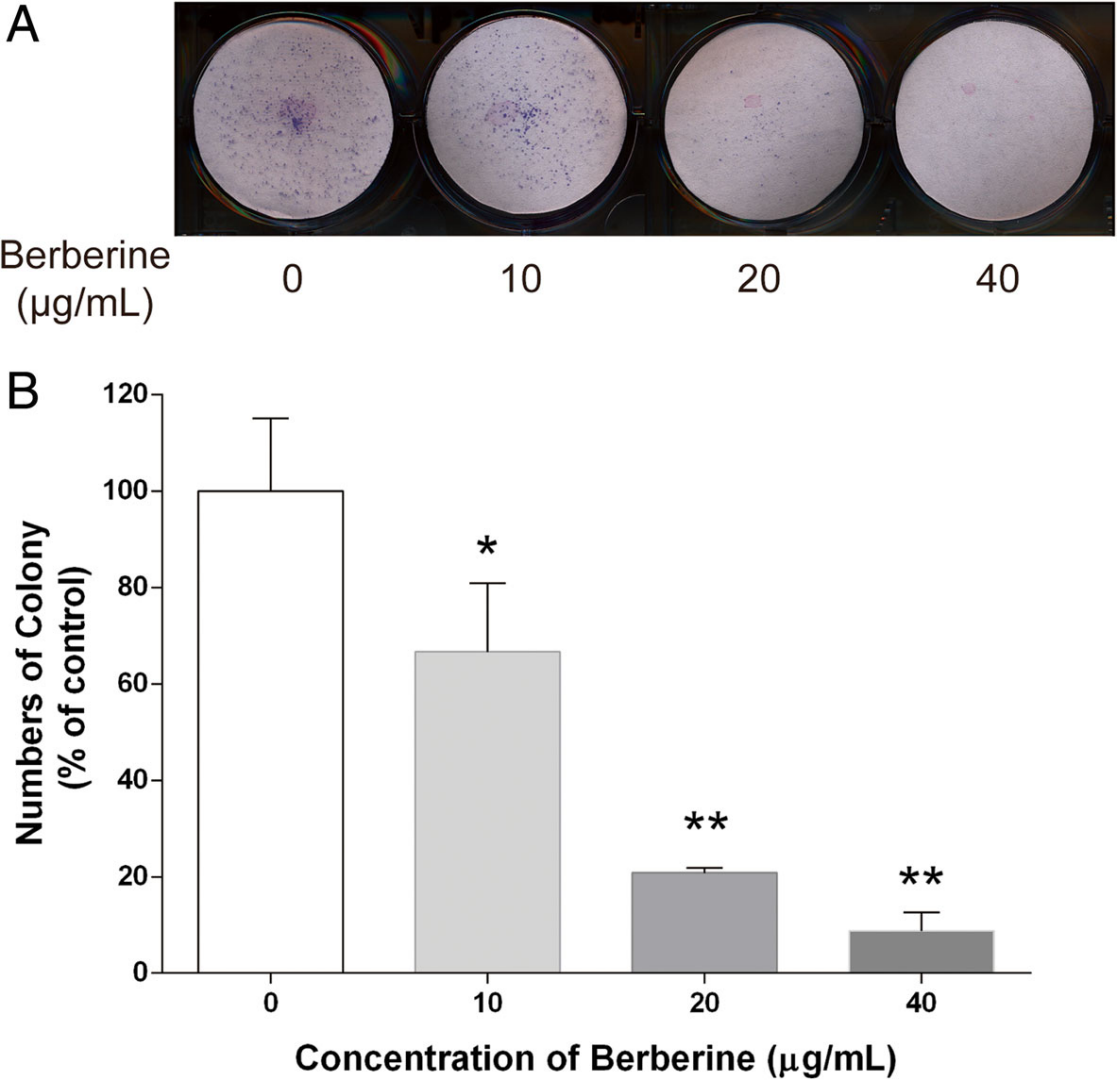
Effect of BBR on colony formation potential of MDA-MB-231 cells. Cells were seeded in 6-well plates at 1000 cells per well following treatment with indicated concentrations of BBR for 24 h. The medium was then replaced with fresh media, and the cells were allowed to grow for 12 days at 37 °C in a humidified atmosphere (5% CO_2_ in air) before staining with Giemsa stain solution. **a** Typical results of the clonogenic assay. **b** The surviving fraction of treated MDA-MB-231 cell line is presented as mean ± S.D. (*n* = 3) of three independent experiments. * *p* < 0.05, ** *p* < 0.01, compared to the control

The ability of cultured cancer cells to proliferate and divide into groups to form colony was considered to have the potential of causing cancer. To explore whether exposure to BBR could suppress the surviving fraction of MDA-MB-231 cells, colony formation assay was conducted. Our results demonstrated that treatments with 10, 20, and 40 μg/ml of BBR significantly reduced the colony numbers of MDA-MB-231 cells by 33.35, 79.23, and 91.24%, respectively (Fig. 2a, b).

### BBR suppresses the migration of MDA-MB-231 cells

**Fig. 3:**
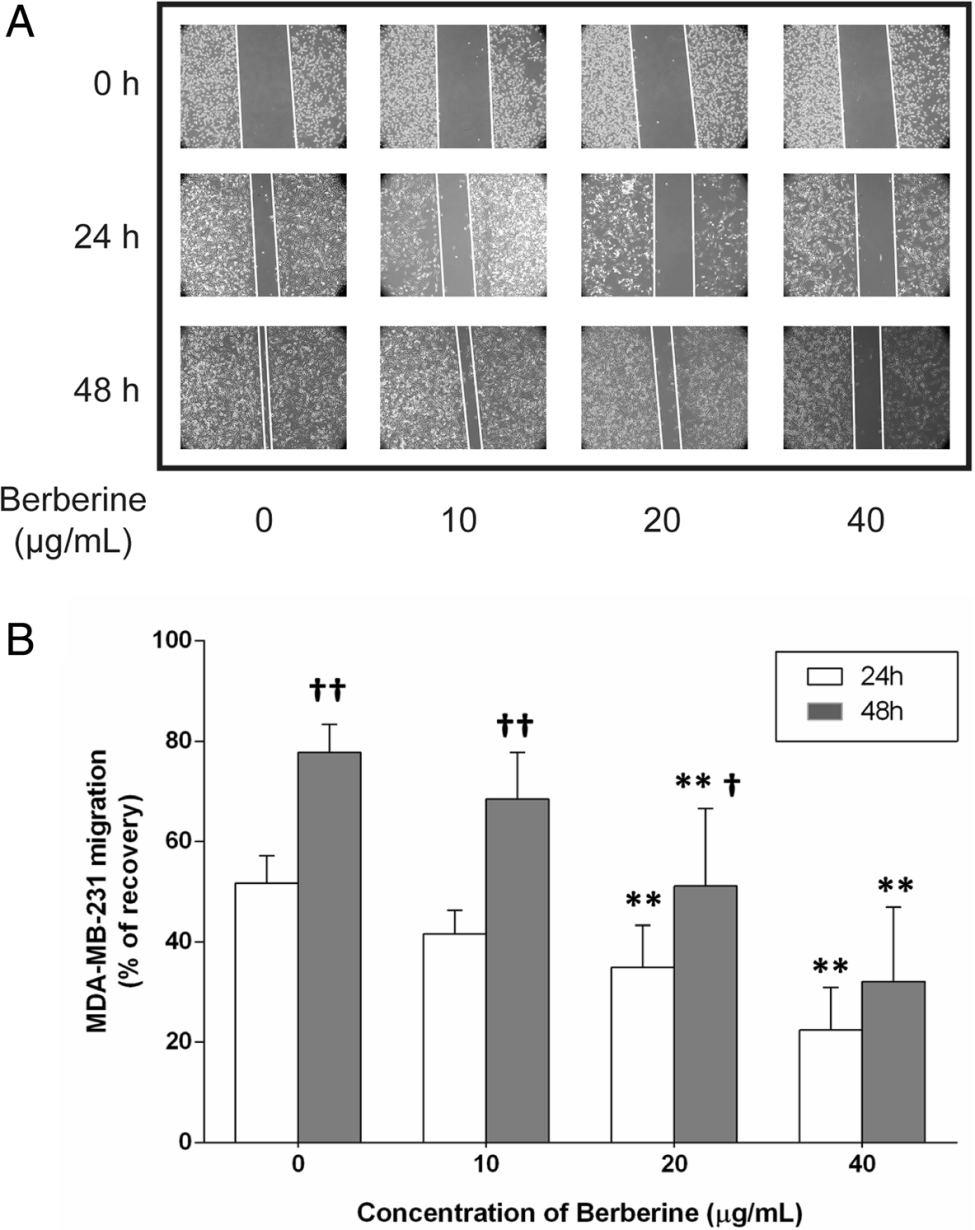
Effect of BBR on MDA-MB-231 cell migration. Cells were seeded into 12-well plates at 1 × 10^5^ cells per well and cultured to near confluence. The wounded monolayer was incubated in culture medium containing various concentrations of BBR for 24 and 48 h. **a** Representative images of wound healing assays for cells treated with various concentrations of BBR, for 24 and 48 h. **b** The percent recovery as determined in the scratch wound healing assay. Data are presented as the mean ± S.D. (*n* = 3) of three independent experiments. *, *p* < 0.01, compared to the control; †, *p* < 0.05, †† *p* < 0.01, compared to 24 h, respectively

We next examined the effect of BBR on cell migration by performing wound healing assay. Our results showed the gaps scraped by a 20-200 μl micropipette tip that remain unfiled by the migrated cells in the 20 and 40 μg/ml BBR-treated groups were significantly wider than those of the untreated group at 24 h (34.98 ± 8.31% and 22.41 ± 8.52%) and 48 h (51.10 ± 15.45% and 32.12 ± 14.84%), furthermore, the gaps in the control group as well as in the 10 and 20 μg/ml BBR-treated groups at 48 h (68.48 ± 9.33% and 51.10 ± 15.45%) were significantly wider than those at 24 h (41.60 ± 4.72% and 34.98 ± 8.31%), respectively (Fig. 3a, b).

### BBR reduces secretion of inflammatory cytokines from MDA-MB-231 cells

**Fig. 4:**
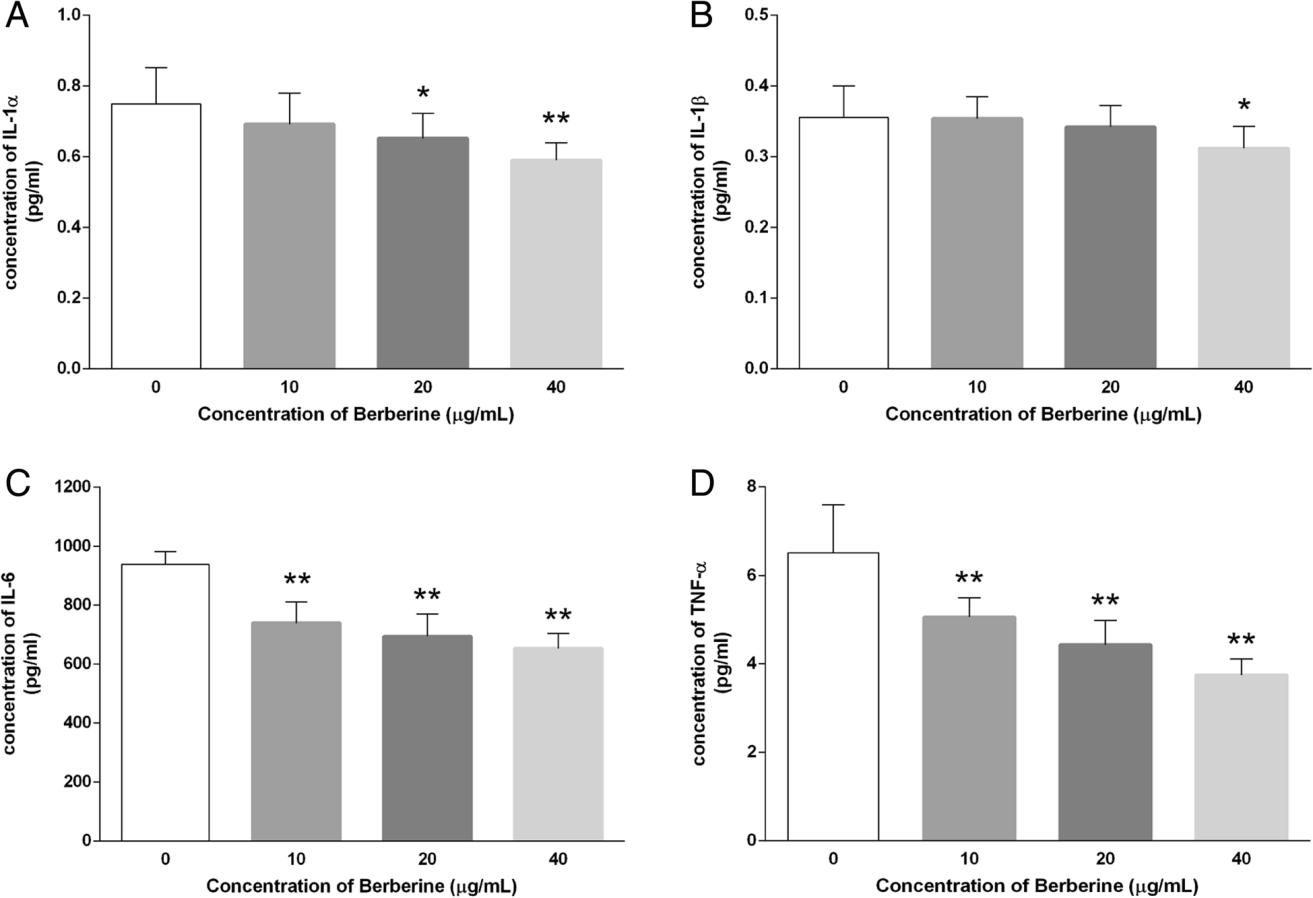
Effect of BBR on inflammatory cytokines in the supernatant of MDA-MB-231 cells. Cells were seeded into 12-well plates at 1 × 10^5^ cells per well following treatment with indicated concentrations of BBR for 48 h, and the supernatant of MDA-MB-231 cells was collected and the cytokines (**a**) IL-1α, (**b**) IL-1β, (**c**) IL-6, and (**d**) TNF-α were analyzed. Data were presented as mean ± S.D. of six duplicated wells. * *p* < 0.05, ** *p* < 0.01, compared to the control

Concentrations of inflammatory cytokines secreted into the supernatant reflected the extent of the inflammatory response. After treatment with various concentrations of BBR (0, 10, 20, 400 μg/ml) for 48 h, the supernatant was collected and analyzed by the Cytokine Magnetic Bead assay to examine whether the treatments affect the release of inflammatory cytokines. As shown in Fig. 4, the level of IL-1α (0.75 ± 0.10 VS 0.69 ± 0.09, 0.65 ± 0.07 and 0.59 ± 0.05 pg/mL), IL-1β (0.36 ± 0.04 VS 0.35 ± 0.03, 0.34 ± 0.03 and 0.31 ± 0.03 pg/mL), IL-6 (938.9 ± 43.26 VS 739.1 ± 71.88, 693.8 ± 75.63 and 652.4 ± 51.13 pg/mL), and TNF-α (6.51 ± 1.08 VS 5.06 ± 0.43, 4.43 ± 0.56 and 3.74 ± 0.36 pg/mL) in the supernatant of MDA-MB-231 cells were reduced in a dose-dependent manner, respectively, indicating the suppressive effect by BBR treatment against excessive secretion of inflammatory cytokines.

### BBR regulates the expression profile of NLRP3 inflammasome related gene in MDA-MB-231 cells

**Fig. 5:**
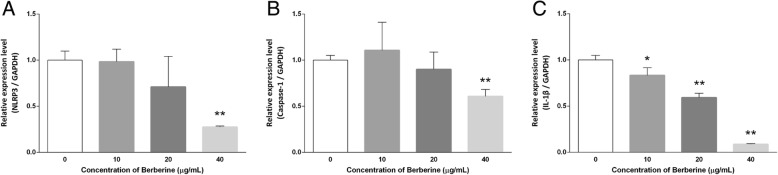
Expression profile of NLRP3 inflammasome related gene in MDA-MB-231 cells treated with BBR. After treatment with various concentrations of BBR for 48 h, the expression profile of NLRP3 inflammasome related gene, (**a**) NLRP3, (**b**) Caspase-1, and (**c**) IL-1β, was analyzed using real-time PCR. Results are shown as the mean ± S.D. from three independent experiments. Significant difference between control and treatment with BBR are shown, * *p* < 0.05, ** *p* < 0.01

Since targeting the NLRP3 inflammasome pathway has been suggested to be a potential strategy for cancer immunotherapy [3, 10, 11, 14], gene expression of related targets in the NLRP3 cascade was detected by real-time PCR to clarify the mechanisms underlying this signaling pathway in MDA-MB-231 cells after treatment with BBR (0, 10, 20, 400 μg/ml) for 48 h. As shown in Fig. 5, the expression profile of NLRP3 inflammasome related gene, NLRP3 mRNA (1.00 ± 0.10 VS 0.98 ± 0.14, 0.71 ± 0.33 and 0.27 ± 0.01), Caspase-1 mRNA (1.00 ± 0.05 VS 1.11 ± 0.31, 0.90 ± 0.19 and 0.61 ± 0.08), and IL-1β mRNA (1.00 ± 0.05 VS 0.83 ± 0.08, 0.59 ± 0.05 and 0.09 ± 0.01), were significantly suppressed in a dose-dependent manner, and glyceraldehyde-3-phosphate dehydrogenase (GAPDH) was served as an endogenous control to normalize the expression. Results were representative of three biological replicates and three technical duplications.

### BBR modulates the NLRP3 inflammasome signaling in MDA-MB-231 cells

**Fig. 6:**
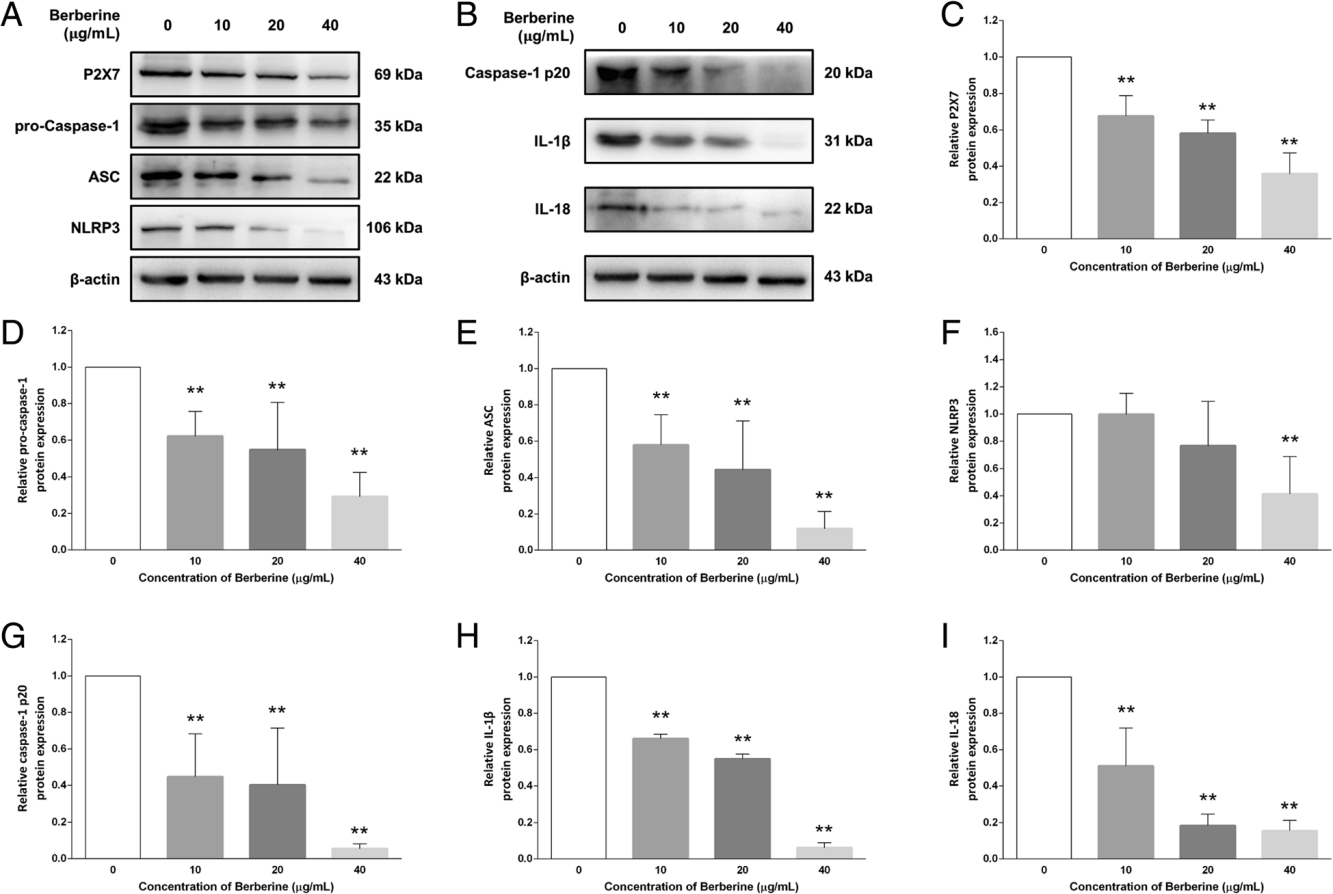
Effects of BBR on the expression profile of NLRP3 inflammasome signaling proteins in MDA-MB-231 cells. After treatment with various concentrations of BBR for 48 h, the protein bands (**a**) and (**b**), as well as expression profile of NLRP3 inflammasome signaling proteins, (**c**) P2X7, (**d**) pro-caspase1, (**e**) ASC, (**f**) NLRP3, (**g**) caspase-1 p20, (**h**) IL-1β and (**i**) IL-18, was analyzed using western blot as described in Material and methods. Significant difference between control and treatment with BBR are shown, * *p* < 0.05, ** *p* < 0.01. Representative image of the expression profile of each protein is shown from three independent experiments

Consistent with that observed in the gene expression profile, after treatment with various concentrations of BBR (0, 10, 20, 400 μg/ml) for 48 h, the expression level of P2X7, considered as sensor of cell damage and a trigger of the NLRP3 inflammasome [38], was significantly down-regulated by BBR in a dose-dependent manner (1.00 ± 0.00 VS 0.68 ± 0.11, 0.59 ± 0.08 and 0.36 ± 0.12). Subsequently, the expressions of major components of NLRP3 inflammasome complex, NLRP3 (1.00 ± 0.00 VS 0.99 ± 0.15, 0.77 ± 0.33 and 0.41 ± 0.28), pro-caspase-1 (1.00 ± 0.00 VS 0.62 ± 0.14, 0.55 ± 0.26 and 0.29 ± 0.13) and ASC (1.00 ± 0.00 VS 0.58 ± 0.16, 0.44 ± 0.27 and 0.12 ± 0.10), were remarkably down-regulated by BBR. Furthermore, the expressions of caspase-1 p20 (1.00 ± 0.00 VS 0.45 ± 0.23, 0.40 ± 0.31 and 0.05 ± 0.03), IL-18 (1.00 ± 0.00 VS 0.51 ± 0.21, 0.18 ± 0.06 and 0.15 ± 0.06) and IL-1β (1.00 ± 0.00 VS 0.66 ± 0.02, 0.55 ± 0.03 and 0.06 ± 0.03) proteins were also decreased after treating with BBR (Fig. 6). These results indicated that BBR probably inhibits P2X7-mediated NLRP3 inflammasome activation.

## Discussion

It has been reported that BBR, single use or in combination with other compound, contributes to the inhibition of breast cancer cells, and the mechanism mainly focus on inducing apoptosis though mitochondrial or caspase-dependent pathway [29, 39–42]. The main purpose of present work was to investigate whether other mechanism, especially the anti-inflammatory effect, was involved in the anti-tumor activity of BBR. Our results clearly indicated that BBR dose-dependently reduce the viability and increase the LDH leakage of MDA-MB-231 cells after 48 h exposure (Fig. 1a, b). In addition, inhibition of colony formation potential and migration of the cells by treatments with different concentrations of BBR have also been observed in this study (Figs. 2 and 3), these results were in the tendencies consistent with previous studies about anti-tumor activity of BBR on the same TNBC cell line [39, 40], although there was slightly difference in the efficacy of BBR. Our results suggested that BBR can effectively affect both tumor proliferation and spontaneous metastasis, and the mechanism is necessarily to be further explored.

In recent years, inflammation has been reported as one of the major risk factors for the development and metastatic process of breast cancer [7, 8], thus anti-inflammation treatment should be a novel strategy against breast cancer. It has been reported that treatment with nonsteroidal anti-inflammatory drugs (NSAIDs), including aspirin and ibuprofen, is bound up with a reduced risk of breast cancer, though the biological mechanisms remain to be elucidated [43]. Celecoxib, a cyclooxygenase-2 (COX-2) selective inhibitor, also exerted anti-tumor effect in primary breast cancer tissue during a clinical trial [44]. As one of the most classical anti-inflammatory agents originated from Chinese Medicine, and although the anticancer effects have been proven, however, the insight mechanisms of BBR in inhibiting inflammatory response were still not well known on breast cancer. Therefore, we then focused on the effect of BBR on the release of inflammatory cytokines and the expression of proteins and mRNAs in the NLRP3 Inflammasome signaling pathways in triple-negative breast cancer cell line MDA-MB-231.

Uncontrolled and sustained generation of cytokines may affect the growth, differentiation and apoptosis of cells [9]. Both IL-6 and TNF-α are considered to be the best characterized tumorigenic cytokines that involved in the promotion, progression and metastasis of tumor [45], and also, co-expression of these two cytokines determines the extension and outcome of breast cancer [46]. The IL-1 family was reported to preferentially express in TNBC and be involved in the development of breast cancer, while inhibition of interleukin 1 receptor (IL-1R) affected the proliferation, prevented the tumor progression and metastasis [47, 48]. More critically, IL-1β is the product of self-activated caspase-1 derived from NLRP3 inflammasome formation. Our results demonstrated that BBR caused a marked reduction in the secretion of proinflammatory cytokines that implicated in carcinogenesis, such as IL-1α, IL-1β, IL-6, and TNF-α, from MDA-MB-231 cells (Fig. 4), indicating that BBR treatment inhibited the maturation and secretion of cytokines and changed the tumor microenvironment.

Besides, a down-regulated behavior was observed with the expression of mRNAs and proteins in the inflammasome cascade in MDA-MB-231 cells treated with different concentrations of BBR. The P2X7 receptor (P2X7R), trigger of the NLRP3 inflammasome, was pivotal in the cancer cell invasion associated with metastasis; thereupon by antagonizing the P2X7R specifically could inhibit the invasiveness of human cancer cell [49]. In previous studies [32], BBR was reported to interfere with the P2X7 signaling in a methionine- and choline-deficient diet induced mouse liver injury model. Our results found that BBR could significantly down-regulate the expression of P2X7 in MDA-MB-231 cells (Fig. 6, a and c), confirming its ability in inhibiting the formation of inflammasome as well as tumor metastasis. Several studies have elucidated the relationship between NLRP3 inflammasome signaling and carcinogenesis [15, 50], that NLRP3 inflammasome inhibition is responsible for cancer prevention. Our results revealed that the mRNA and protein expression of Nod-like receptor protein NLRP3, ASC and pro-caspase-1, components in the multiprotein platform of inflammasome, were down-regulated after BBR treatment (Figs. 5 a and 6, a, d, e and f), and then directly resulted in the decreased activity of caspase-1 p20 and low expression of IL-1β and IL-18 (Figs. 5 b, c and 6, b, g, h and i). These findings suggested the BBR treatment associated with inhibiting the NLRP3 inflammasome signaling pathway and prevent the process of tumor development at both the mRNA and protein levels.

## Conclusion

In conclusion, we confirmed the cytotoxic capability of BBR on TNBC MDA-MB-231, manifested as decreased cell growth, LDH releasing, cloning formation ability. Concomitantly, the migration capability of MDA-MB-231 cells was decreased by BBR. More critically, we identified a new potential mechanism associated with the NLRP3 inflammasome pathway inhibition. Our current study revealed that BBR may exert the inhibition of cell growth and migration in breast cancer associated with the regulation of inflammasome pathway, and could therefore have potential clinical therapeutic relevance. However, the definite mechanism of BBR on breast cancer should be further investigated.

## Data Availability

The datasets used and/or analyzed during the current study available from the corresponding author on reasonable request.

## Abbreviations

AMPK: 5′ AMP-activated protein kinase
ASC: Apoptosis-associated speck-like protein containing a caspase-activation and recruitment domain
BBR: Berberine
COX-2: Cyclooxygenase-2
DMEM: Dulbecco’s Modified Eagle Medium
FBS: Fetal bovine serum
GAPDH: Glyceraldehyde-3-phosphate dehydrogenase
IBD: Inflammatory bowel disease
IL-18: Interleukin-18
IL-1α: Interleukin-1α
IL-1β: Interleukin-1β
IL-6: Interleukin-6
LDH: lactate dehydrogenase
mTOR: Mechanistic target of rapamycin
NLRP3: Nucleotide-binding oligomerization domain-like receptor containing pyrin domain 3
NSAIDs: Nonsteroidal anti-inflammatory drugs
ox-LDL: Oxidized low-density lipoprotein
P2X7: P2X purinoceptor 7
PBS: Phosphate-buffered saline
PCR: Polymerase chain reaction
TNBC: Triple-negative breast cancer
TNF-α: Tumor necrosis factor-α
